# Insulin-like growth factor-I serum levels and their biological effects on *Leishmania* isolates from different clinical forms of American tegumentary leishmaniasis

**DOI:** 10.1186/s13071-016-1619-x

**Published:** 2016-06-11

**Authors:** Luana Dias de Souza, Célia Maria Vieira Vendrame, Amélia Ribeiro de Jesus, Márcia Dias Teixeira Carvalho, Andréa Santos Magalhães, Albert Schriefer, Luiz Henrique Guimarães, Edgar Marcelino de Carvalho, Hiro Goto

**Affiliations:** Laboratório de Soroepidemiologia e Imunobiologia, Instituto de Medicina Tropical de São Paulo, Universidade de São Paulo, Avenida Dr. Enéas de Carvalho Aguiar n 470, prédio II, 4 andar, CEP 05403-000 São Paulo, SP Brazil; Serviço de Imunologia, Hospital Universitário Professor Edgard Santos, Universidade Federal da Bahia, Salvador, BA Brazil; Laboratório de Biologia Molecular, Departamento de Medicina Interna e Patologia, Universidade Federal de Sergipe, Aracaju, SE Brazil; Departamento de Medicina Preventiva, Faculdade de Medicina, Universidade de São Paulo, Sao Paulo, SP Brazil

**Keywords:** *Leishmania braziliensis*, Tegumentary leishmaniasis, THP-1, IGF-I level

## Abstract

**Background:**

American tegumentary leishmaniasis (ATL) in Brazil is mostly caused by *Leishmania* (*Viannia*) *braziliensis*, with known forms of the disease being cutaneous (CL), mucosal (ML) and disseminated (DL) leishmaniasis. The development of the lesion in ATL is related both to the persistence of the *Leishmania* in the skin and to the parasite-triggered immune and inflammatory responses that ensue lesions. In this context one factor with expected role in the pathogenesis is insulin-like growth factor (IGF)-I with known effects on parasite growth and healing and inflammatory processes. In the present study, we addressed the effect of IGF-I on intracellular amastigote isolates from CL, ML and DL patients within human macrophage and we evaluated the IGF-I and IGF-binding protein-3 (IGFBP3) serum levels in patients presenting different clinical forms and controls from the endemic area.

**Methods:**

We evaluated biological variability in the responses of intracellular amastigotes of *Leishmania* isolates derived from CL, ML, and DL patients from an area for ATL in response to IGF-I. Intracellular amastigote growth was evaluated using the human macrophage cell line THP-1. Arginase activity in infected cells was evaluated quantifying the generated urea concentration. Serum samples from patients and controls were assayed using chemiluminescent immunometric assay to determine IGF-I and IGFBP3 levels.

**Results:**

We observed an increase in intracellular parasitism upon IGF-I stimulus in 62.5 % of isolates from CL, in 85.7 % from ML and only 42.8 % from DL cases. In DL, the basal arginase activity was lower than that of CL. We then evaluated the IGF-I and IGFBP3 serum levels in patients, and we observed significantly lower levels in ML and DL than in CL and control samples.

**Conclusions:**

The data suggest that IGF-I is modulated distinctly in different clinical forms of tegumentary leishmaniasis. IGF-I seemingly exerts effect on parasite growth likely contributing to its persistence in the skin in earlier phase. In addition the decreased IGF-I serum levels may affect the modulation of inflammation and lesion healing in chronic phase. In view of potential role of IGF-I in the pathogenesis of ATL we can speculate on therapeutic procedures taking into account the local IGF-I level.

## Background

Human leishmaniasis caused by *Leishmania* (*Viannia*) *braziliensis* infection has a broad spectrum of manifestations ranging from an asymptomatic infection to severe destructive ulcerated and inflammatory forms. The main clinical forms are cutaneous leishmaniasis (CL), severe destructive mucosal leishmaniasis (ML) and disseminated leishmaniasis (DL) [1]. Some studies relate the different disease manifestations to the intraspecific variability of *L*. (*V*.) *braziliensis* not only in Brazil but also in Colombia [2–4] but not in another study [5]. Besides, the presence of *Leishmania* RNA virus 1 in *L*. (*V*.) *braziliensis* has been associated with the development of ML but not in all ML cases [6]. Therefore, the wide spectrum of manifestations caused by *L*. (*V*.) *braziliensis* are still poorly understood.

It is known that the development of the lesion in tegumentary leishmaniasis is related both to the persistence of the *Leishmania* in the skin and to the parasite-triggered immune and inflammatory responses that result in lesion development [7, 8]. The Th1-type immune response controls the parasite growth [7] but in chronic and severe lesions such as in mucosal leishmaniasis maintenance of the supposedly beneficial immune response turn into chronic and harmful process [9, 10].

In this context one of elements that may have role in the pathogenesis is insulin-like growth factor (IGF)-I since this factor has effect on both parasite growth [11] contributing to its persistence and on the healing and inflammatory processes [12–14].

We have been studying the effect of IGF-I on *Leishmania* and in leishmaniasis, demonstrating its impact on parasite growth and lesion development. IGF-I in cases of *L*. (*L*.) *amazonensis* showed effects increasing the parasite growth and cutaneous leishmaniasis lesion development [11]. In *Leishmania*-macrophage interaction, IGF-I exerts effect on *Leishmania* and host cells, mainly inducing arginase expression and activity, leading to alternative activation of the macrophages interfering with inducible nitric oxide synthase expression [11, 15, 16]. Because biological effects may differ in other species and American tegumentary leishmaniasis is mostly caused by *L*. (*V*.) *braziliensis*, we analyzed the effect of this growth factor on this parasite species. Then, we observed complex results related to the source of the parasite isolates. IGF-I induced higher basal arginase activity in promastigote isolates derived from patients with CL and DL; however, arginase activity was already increased under basal conditions in isolates from ML with no further increase upon IGF-I stimulus [17]. In the present study, we addressed the effect of IGF-I on amastigote forms of isolates from CL, ML and DL patients within the THP-1 human macrophage lineage. We used the macrophage lineage rather than macrophages derived from peripheral blood monocytes to avoid variations in cellular response in different individuals. We observed an increase in intracellular parasitism upon IGF-I stimulus in 62.5 % of isolates from CL, in 85.7 % from ML and only 42.8 % from DL cases. Although we observed differences among *Leishmania* isolates, they do not clearly explain their different clinical manifestations.

Since in addition to the effect on parasite growth, IGF-I has a pleiotropic effect on cell migration [18], wound healing [12, 13] and inflammatory process [14], in the present study, we evaluated the IGF-I and IGF-binding proteins-3 (IGFBP3) serum levels in patients presenting different clinical forms, CL, ML and DL and controls from the endemic area. In this analysis, both IGF-I and IGFBP3 levels decreased in ML and DL compared with CL and controls. The data as a whole suggest that IGF-I is modulated in different clinical forms of tegumentary leishmaniasis, and IGF-I seemingly exerts effects on both parasite growth likely contributing to its persistence in the skin and its lower level affecting the modulation of inflammation and lesion healing process. In view of potential role of IGF-I in pathogenesis of tegumentary lesion development we can speculate on therapeutic procedures considering local IGF-I level.

## Methods

### Study design and subjects

The patients included in this study were from Corte de Pedra municipality, Bahia state, Northeastern Brazil, an area with endemic tegumentary leishmaniasis mostly caused by *L*. (*V*.) *brasiliensis*. The patients were approached at the Corte de Pedra Health Post from 2002 through 2007. All participants were volunteers and provided their individual informed consent.

A leishmaniasis diagnosis was made based on a clinical feature of these forms of leishmaniasis along with one of the following criteria: parasite isolation or a positive skin test for *Leishmania* soluble antigen and the presence of histopathological findings suggestive of leishmaniasis. Patients with CL had a typical ulcerative lesion in the skin; ML patients had a metastatic mucosal nasal lesion that was not contiguous with the primary cutaneous lesion, and DL patients had polymorphic lesions (acneiform, papular, nodular and/or ulcerated) on two or more parts of their body. The exclusion criteria included HIV infection, diabetes mellitus and pregnancy. All patients were evaluated before receiving therapy and during the active disease. The ages ranged from 21 through 65 years. One-hundred fourteen patients were studied, suffering from CL (*n* = 65), ML (*n* = 20), DL (*n* = 29) and 14 endemic control subjects living in the same municipality. The sera were stored at -70 °C until they were analyzed.

### Isolates of *L*. (*V*.) *braziliensis*

The 22 isolates of *Leishmania* were obtained from patients presenting with the following different clinical forms of the disease: cutaneous leishmaniasis (*n* = 8), mucosal leishmaniasis (*n* = 7) and disseminated leishmaniasis (*n* = 7). The isolates were obtained by aspirating the lesion, and the samples were grown in tubes with biphasic medium (LIT/NNN) supplemented with 10 % heat inactivated fetal calf serum (FCS) (Cripion Biotechnology, Brazil) at 26 °C. The parasite isolates were cryopreserved and expanded in Schneider’s insect medium (Sigma), pH 7.2, supplemented with 10 % FCS. Most of the isolates were characterized as *L*. (*V*.) *braziliensis* by isoenzyme electrophoresis and monoclonal antibodies in the Departamento de Bioquimica e Biologia Molecular, Instituto Oswaldo Cruz, FIOCRUZ, Rio de Janeiro, Brazil [19]. The promastigotes used in the experiments were in the stationary phase of growth and had no more than four passages in culture.

### THP-1 monocytic cell line and cell culture

The monocytic cell line THP-1 (ATCC) was grown and replicated every 4 days in RPMI 1640 supplemented with 5 % FCS, 100 UI/ml streptomycin, 2 mM L-glutamine, 11 mM sodium bicarbonate and maintained in a humidified atmosphere at 37 °C with 5 % CO2. The viability was assessed by a dye exclusion test using 0.02 % trypan blue in phosphate-buffered saline 0.01 M, pH 7.2 (PBS); to test for infection, 2 × 10^5^ cells in 500 μl in RPMI 1640 with 2 % FCS were distributed in each well of 24-well culture plates in triplicate with sterile, round 13 mm coverslips.

THP-1 monocytes were submitted to 20 ng/ml phorbol 12-myristate 13 acetate (PMA) for 24 h at 37 ° C, 5 % CO_2_ for differentiation into macrophages [20]. The non-adherent cells were removed, the medium was replaced, and the cells were maintained for 24 h in the same conditions. Infection with *Leishmania*/macrophage (ratio of 10:1), using isolates from CL, ML and DL of *L*. (*V*.) *braziliensis*, was performed with or without stimulation with IGF-I (50 ng/ml) and incubated at 33 °C, 5 % CO_2_. After 4 h, the plates were washed with warm PBS to remove non-internalized parasites, and the RPMI 1640 medium with 2.0 % FCS and IGF-I was replaced. After different incubation times, the supernatant was collected and stored at -70 °C for evaluation of arginase activity, and the coverslips were taken for intracellular parasite counting.

### Evaluation of intracellular parasite load

The coverslips removed from the plates were stained with Giemsa dyes, and intracellular parasites were counted under a light microscope (Carl Zeiss, Germany). For each experimental condition, 600 cells and intracellular parasites were counted by two independent observers, blinded for experimental conditions. The data are presented as the number of parasites per 100 cells.

### Arginase activity

The cells and promastigotes were removed from the cultures, lysed and submitted to arginase activity determination [21]. Briefly, to activate the arginase, 50 μl of lysate was treated with the same volume of 5 mM MnCl_2_, 25 mM Tris-HCl pH 7.4 at 56 °C for 10 min. Then, 25 μl of 0.5 M L-arginine pH 9.7 was added to 25 μl of the activated lysate and incubated at 37 °C for 60 min. The reaction was stopped with 400 μl of H_2_SO_4_/H_3_PO_4_/H_2_O (1/3/7, v/v/v). The urea concentration was measured at 540 nm in a spectrophotometer Multiskan MCC/340 P version 2.20 plate reader (Labsystems, Vantaa, Finland) after the addition of 25 μl of 9 % α-isonitrosopropiophenone in 100 % methanol and submitted to 100 °C for 45 min. One unit of enzyme activity was defined as the amount of enzyme that catalyzes the formation of 1 μmol of urea per minute.

### Analyzes of circulation IGF-I and IGFBP3 levels

IGF-I and IGFBP3 assays were performed using the IMMULITE® 2000 (DPC-Diagnostics Products Corporation, Los Angeles, CA, USA). This procedure is an automated 2-site sandwich immunoassay with a chemiluminescent immunometric assay. It was configured and calibrated on an instrument at Laboratório de Hormônio do Hospital das Clínicas da Universidade de São Paulo, according to the manufacturer’s instructions.

### Statistical analysis

Statistical analysis was performed using GraphPad Prism5 (GraphPad Software, Inc., San Diego, CA, USA). The isolates results were submitted to ANOVA with *post-hoc* Student-Newman Keuls test. An alpha error of 5 % (*P* = 0.05) was considered to determine statistical significance. The IGF-I and IGFBP3 assay results were expressed as medians and percentiles (25–75) and submitted to the Kruskal-Wallis test with the Student Newman-Keuls *post-hoc* test to statistically compare the groups.

## Results

In this study, we initially evaluated the parasitism and the arginase activity of the isolates of *L*. (*V*.) *braziliensis* derived from patients presenting CL (*n* = 8), ML (*n* = 7) or DL (*n* = 7).

We evaluated the parasites as amastigotes within human monocytic cell line THP-1 in the presence or the absence of the growth factor IGF-I. This cell line can be activated by IGF-I [22]. Having perceived differences in the biological parameters in this approach, we then evaluated the IGF-I and IGFBP3 serum levels in patients and in controls living in the same endemic area.

**Fig. 1:**
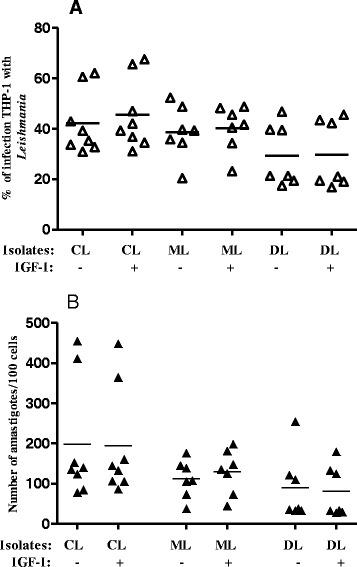
Effect of IGF-I on parasitism in THP-1 with isolates of *Leishmania* from patients with cutaneous (CL, *n* = 8), mucosal (ML, *n* = 7) or disseminated (DL, *n* = 7) forms of the disease. THP-1 cells infected with the isolates of *Leishmania* (*V*.) *braziliensis* were stimulated or not with IGF-I (50 ng/ml) throughout the culture period of 48 h. Parasitism was evaluated by optical microscopy and the result is represented as the number of parasites per 100 cells (**a**). Parasite load ratio upon IGF-I-stimuli and basal conditions in THP-1 cells infected with *Leishmania* (**b**). Horizontal bar represents the mean value. Results representative of two experiments with each isolate tested in triplicate

The percentage of infected cells in basal conditions (i.e. no IGF-I stimulus) indicated that the parasitism tended to be lower with isolates of *Leishmania* coming from DL than from CL and ML cases (Fig. 1a). The parasite load (number of amastigotes/100 infected cells) in the basal conditions tended to be greater with parasites from CL than ML and DL cases (Fig. 1b and Table 1). Evaluating the effect of IGF-I on the isolates as a whole in each group, we observed no significant differences in the percentage of infection (Fig. 1a) or in the number of amastigotes/100 cells (Fig. 1b and Table 1).

**Table 1:** Effect of IGF-I on parasitism in THP-1 with isolates of *Leishmania*

*Leishmania* from	CL		ML		DL	
IGF-I (50 ng/ml)	–	+	–	+	–	+
Parasite isolates	124	107	139	199	255	180
	78	87	146	148	110	133
	449	455	73	73	33	33
	140	145	177	182	36	28
	135	161	108	126	122	125
	412	365	38	45	33	34
	85	106	106	136	38	30
	153	133				

THP-1 cells were infected with the isolates of *L*. (*V*.) *braziliensis* from patients with cutaneous (CL, *n* = 8), mucosal (ML, *n* = 7) or disseminated (DL, *n* = 7) forms of the disease and stimulated or not with IGF-I (50 ng/ml) throughout the culture period of 48 h. Parasitism was evaluated by optical microscopy and the result is represented as the number of parasites per 100 cells. Results representative of two experiments with each isolate tested in triplicate

**Fig. 2:**
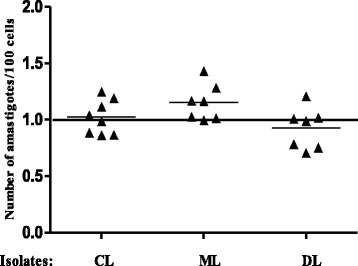
Infection ratio of THP-1 cells infected with *Leishmania* isolates from patients with different clinical presentations upon IGF-I stimulus. Infection ratio between parasite load of cells upon IGF-I stimulus and basal infection without stimulus. THP-1 cells were infected with *Leishmania* isolates from CL (*n* = 8), ML (*n* = 7) or DL (*n* = 7) and maintained in culture with or without IGF-I (50 ng/ml) for 48 h. Parasitism was assessed by optical microscopy and the results expressed as number of amastigotes/100 cells

However, to further assess the effect of IGF-I in infections and have a better view of the behavior of individual isolates, we calculated the ratio of the individual parasite load in cultures stimulated with IGF-I in relation to its basal culture in which a ratio greater than 1.0 would indicate the positive effect on parasite growth (Fig. 2). After a culture period of 48 h, we observed an increase in the parasitism ratio upon IGF-I stimulus in 62.5 % (5 of 8) of the isolates from CL cases, in 85.7 % (6 of 7) of the isolates from ML cases and in only 42.8 % (3 of 7) from DL patients. Notably, the isolates from the DL cases showed reduced growth in the presence of IGF-I.

**Fig. 3:**
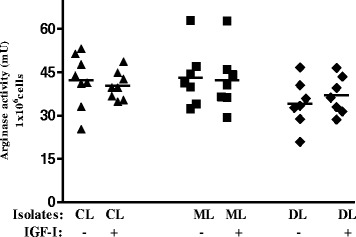
Effect of IGF-I on arginase activity in THP-1 infected with *Leishmania* isolated from patients with different clinical presentations. THP-1 cells were infected with *Leishmania* isolates from CL (*n* = 8), ML (*n* = 7) or DL (*n* = 7) and maintained in culture with or without IGF-I (50 ng/ml) for 24 h. Representative result of two experiments (mean ± standard deviation). One unit of arginase activity = the amount of enzyme that catalyzed the formation of 1 micromol urea/min. Result is shown as: mU arginase/1 × 10^6^ cells)

In the analysis of arginase activity, both basal and IGF-I-stimulated levels were lower in those cells infected with isolates from DL than in those from CL and ML (Fig. 3).

The above results suggest that IGF-I is beneficial or deleterious depending on the isolates; therefore, we analyzed whether IGF-I serum levels were altered in leishmaniasis patients presenting with different clinical forms of the disease.

The clinical features of the studied patients are presented in Tables 2 and 3. There were no significant differences in the group distributions according to age (Table 2) and sex (Table 3). The higher percentage of men than women in all groups of patients likely reflects the epidemiological characteristics of the transmission of infection with higher exposure of the male to the insect bite due to their agricultural activity, although sex-related biological factors cannot be discarded.

**Table 2:** Clinical profile of study patients with Leishmaniasis

	Clinical Form			
Parameters	Cutaneous L	Mucosal L	Disseminated L	Controls
Number	65	20	29	14
Age (years)	34 ± 10	43 ± 10	38 ± 12	30 ± 8

The age was expressed as mean ± standard deviation. See Methods for further details. ANOVA test with the Student Newman-Keuls *post-hoc* test for statistical comparison among groups. *P* > 0.05

**Table 3:** Gender distribution of American Tegumentary Leishmaniasis (ATL) patients

Clinical Form	Sex		OR	95 % CI	*P*-value*
	Male	Female			
Controls^1,2,4^	12	2			
Cutaneous L^1,3,5^	55	10	1.1	0.21–5.63	^1^0.9172
Mucosal L^2,3,6^	13	7	3.2	0.55–18.72	^2^0.1779
			3.0	0.95–9.26	^3^0.0551
Disseminated L^4,5,6^	26	2	0.5	0.06–3.68	^4^0.4572
			0.4	0.09–2.07	^5^0.2768
			0.1	0.02-0.79	^6^0.0148

*P value analyzed by the *χ* ^2^ test. Note: Odds ratios (OR) and *χ* ^2^ tests were performed with groups that bear the same number in superscript; the respective odds ratio, 95 % confidence interval (CI), and *P*-value for each comparison has the same number in superscript. No statistically significant differences were found

**Fig. 4:**
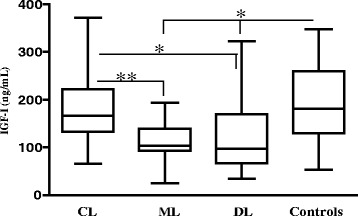
IGF-I serum levels in patients with American Tegumentary Leishmaniasis. Serum of patients with CL (n=65), ML (n=20), DL (n=29), and healthy subjects were measured by chemiluminescent immunometric assay (IMMULITE® 2000-DPC). The horizontal bar represents the median value. Kruskal-Wallis test with the Dunn contrast post-test: *CL in relation to DL; Controls in relation to DL and ML *P* < 0.05; **CL in relation to ML *P* < 0.001

The IGF-I and IGFBP3 serum levels were analyzed, and the fact that the groups do not differ in age distribution was important for these evaluations because these levels vary considerably in different age ranges [23]. While patients with CL showed similar average levels of IGF-I compared with control subjects, patients with ML and DL had significantly lower levels than patients with CL (Fig. 4). These results were similar when analyzing men and women separately (data not shown). In contrast, IGFBP3 levels in the serum of patients were similar between the studied groups (data not shown).

## Discussion

The pathogenesis of American tegumentary leishmaniasis currently is considered multifactorial, due to not only specific immune response [24] but also parasite diversity [4, 17] and other factors related to the host [25, 26]. In this scenario, we have been studying the role of IGF-I in leishmaniasis mainly in vitro and in experimental models of leishmaniasis. The role of IGF-I in human leishmaniasis has not been established.

Based on our previous experimental data, IGF-I can play a role in the progression of *Leishmania* (*Leishmania*) *amazonensis* infection by (i) decreasing NO production in macrophages and allowing parasite multiplication, (ii) modulating immune responses by increasing TGF-β and decreasing IFNγ production in promastigote infected macrophages, and (iii) inducing apoptotic mimicry characterized by exposure of phosphatidyl serine on amastigotes but without leading to death [15, 16]. However, it is unknown whether similar effects of IGF-I would occur in other *Leishmania* species.

Because tegumentary leishmaniasis in Brazil is mostly caused by *L*. (*V*.) *braziliensis* [1] and different biological effects may occur upon IGF-I stimulus, we initially studied its effect on promastigotes. Focusing on the increase of arginase activity of the parasite, which was seen as main effect of IGF-I [16], we confronted more complex outcomes that were dependent on *Leishmania* isolates, whether coming from patients with CL, ML or DL. IGF-I induced higher basal arginase activity in *L*. (*V*.) *braziliensis* promastigote isolates from CL and DL but not in ML. Conversely, in *Leishmania* isolate from ML the arginase activity was already increased in basal conditions [17]. In the present study, we analyzed the effect of IGF-I on intracellular amastigotes in sequence.

The intracellular amastigote growth was evaluated here using human macrophage cell line THP-1 rather than macrophages derived from peripheral blood monocytes to avoid the variation in cellular response that would occur if obtained from different individuals. IGF-I induced an increase in parasitism in CL and ML isolates but a decrease in parasitism in DL isolates. In CL and ML these data suggest that the presence of IGF-I may contribute to the persistence of the parasite in the skin. In DL isolates, basal arginase activity was also lower than that of CL. However, arginase activity was similar with and without IGF-I stimulus, leaving mechanisms that need to be further explored.

To proceed with the study of the involvement of IGF-I in different clinical manifestations, IGF-I and IGFBP3 serum levels were evaluated in patients with CL, ML and DL. In CL, both of these levels were similar to those in healthy controls, while in ML and DL, both levels were decreased. Altogether, these data indicate that IGF-I cannot be interpreted as having similar effects in patients presenting different clinical manifestations and that the effects may encompass healing and inflammatory processes beyond parasite growth.

American tegumentary leishmaniasis is characterized by an intense inflammation with a high production of tumor necrosis factor-α (TNF-α) and interferon-γ (IFN-γ), molecules that are necessary to protect the host against *Leishmania* but can also cause tissue damage even with a scarce number of parasites in the lesions [25, 27]. In the early phase of the disease when patients have a small cutaneous lesion or when is evolving to heal [28], this T helper type 1 immune response is known to be down modulated [29]. IGF-I is known to be increased in the initial phase of skin injury because this molecule is necessary for epidermis maintenance [30, 31], which would occur also in CL. In CL, because IGF-I is suggested by the present findings to have a parasite growth promoting effect on *Leishmania* isolates and has its serum level maintained in the level of controls, IGF-I would probably contribute to the progression of parasite growth and establishment of *Leishmania* in the host skin.

Cutaneous ulcer is the first lesion of tegumentary leishmaniasis that later can be complicated by the appearance of mucosal or disseminated disease. After the initial phase of *L*. (*V*.) *braziliensis* infection, a very strong Th1 immune response occurs that is associated with a decrease in IL-10 production [32]. IFN-γ and TNF-α are also produced in even higher concentrations in ML patients [33], and this increased production of cytokines may relate to the lower level of IGF-I in patients with ML observed in the present study.

In DL patients, a slight decrease in IFN-γ production was observed when analyzing peripheral blood mononuclear cells, which may result from the migration of activated T cells to the multiple lesions and to the mucosal tissue because up to 40 % of DL patients have mucosal disease [34]. In DL, IFN-γ production is still high that may relate to the decreased IGF-I serum levels found here in this form of the disease.

IGF-I was shown to decrease vascular inflammatory process in mouse model of atherosclerosis [14]. Because the pathogenesis of American tegumentary leishmaniasis is based on an exaggerated immune response [35, 36], a decrease in IGF-I in ML and DL may contribute to the persistence of the inflammatory response and further on the delayed healing of the lesion.

Having pathogenic mechanism involving IGF-I in mind we can speculate the use of IGF-I to stimulate healing and control of inflammation in chronic and severe cases of ATL where IGF-I level is decreased. There are studies on IGF-I use in other dermatological lesions. In diabetes mellitus ulcer it is suggested that the healing is delayed due to a decrease in IGF-I level in the skin [12] and some approaches have been tried. In diabetes mellitus patients the skin ulcer healed with an increase in local IGF-I level upon the use of hyperbaric oxygen therapy [37]. In experimental diabetes model local use of IGF-I cream has also improved the healing [38]. In ATL IGF-I may increase the parasite growth but it would be in the tissue environment where strong Th1-type immune response is present and thus the parasite growth would be restrained.

## Conclusions

In the present study, analyzing the effect of IGF-I on amastigotes of *Leishmania* isolates from patients and evaluating the IGF-I serum levels, we observed results that suggest IGF-I affecting both parasites and healing and inflammatory processes with IGF-I being modulated differently in distinct clinical forms of ATL. We suggest that in the initial phase of the disease, there are maintained levels of IGF-I that contribute to downregulation of the immune response and for parasite growth/persistence. In the late phase of the infection, with the decrease in IGF-I levels it would allow mainly the persistence of a tissue damage.

In view of potential role of IGF-I in the pathogenesis of ATL we can speculate on therapeutic procedures taking into account the local IGF-I level.

## Abbreviations

ATCC, American type culture collection; ATL, American tegumentary leishmaniasis; CI, confidence interval; CL, cutaneous leishmaniasis; DL, disseminated leishmaniasis; FCS, fetal calf serum; HIV, human immunodeficiency virus; IFN-γ, interferon gamma; IGF, insulin-like growth factor; IGFBP3, IGF-binding proteins-3; IL-10, interleukin 10; ML, mucosal leishmaniasis; OR, odds ratios; PBS, phosphate-buffered saline; PMA, phorbol 12-myristate 13 acetate; RPMI, Roswell park memorial institute medium; TGF-β, transforming growth factor beta; TNF-α, tumor necrosis factor alpha
